# Therapeutic Strategies Targeting Mitochondrial Calcium Signaling: A New Hope for Neurological Diseases?

**DOI:** 10.3390/antiox11010165

**Published:** 2022-01-15

**Authors:** Laura R. Rodríguez, Tamara Lapeña-Luzón, Noelia Benetó, Vicent Beltran-Beltran, Federico V. Pallardó, Pilar Gonzalez-Cabo, Juan Antonio Navarro

**Affiliations:** 1 Department of Physiology, Faculty of Medicine and Dentistry, Universitat de València-INCLIVA, 46010 Valencia, Spain; luzon.lapena@uv.es (T.L.-L.); noeliabg92@gmail.com (N.B.); vibelbel@alumni.uv.es (V.B.-B.); Federico.V.pallardo@uv.es (F.V.P.); 2 Associated Unit for Rare Diseases INCLIVA-CIPF, 46010 Valencia, Spain; 3 Centro de Investigación Biomédica en Red de Enfermedades Raras (CIBERER), 46010 Valencia, Spain; 4 Department of Genetics, Universitat de València-INCLIVA, 46100 Valencia, Spain; 5 INCLIVA Biomedical Research Institute, 46010 Valencia, Spain

**Keywords:** calcium, mitochondria, endoplasmic reticulum, neurological, sigma-1 receptor, mitochondrial calcium uniporter, amyotrophic lateral sclerosis, Charcot–Marie–Tooth, Friedreich’s ataxia

## Abstract

Calcium (Ca^2+^) is a versatile secondary messenger involved in the regulation of a plethora of different signaling pathways for cell maintenance. Specifically, intracellular Ca^2+^ homeostasis is mainly regulated by the endoplasmic reticulum and the mitochondria, whose Ca^2+^ exchange is mediated by appositions, termed endoplasmic reticulum–mitochondria-associated membranes (MAMs), formed by proteins resident in both compartments. These tethers are essential to manage the mitochondrial Ca^2+^ influx that regulates the mitochondrial function of bioenergetics, mitochondrial dynamics, cell death, and oxidative stress. However, alterations of these pathways lead to the development of multiple human diseases, including neurological disorders, such as amyotrophic lateral sclerosis, Friedreich’s ataxia, and Charcot–Marie–Tooth. A common hallmark in these disorders is mitochondrial dysfunction, associated with abnormal mitochondrial Ca^2+^ handling that contributes to neurodegeneration. In this work, we highlight the importance of Ca^2+^ signaling in mitochondria and how the mechanism of communication in MAMs is pivotal for mitochondrial maintenance and cell homeostasis. Lately, we outstand potential targets located in MAMs by addressing different therapeutic strategies focused on restoring mitochondrial Ca^2+^ uptake as an emergent approach for neurological diseases.

## 1. Introduction

Calcium (Ca^2+^) is the most ubiquitous secondary messenger in intracellular signaling of most living cells, acting as a key connection between extracellular signals and intracellular responses [1]. The most remarkable property of Ca^2+^ is that such a simple bivalent ion is involved in a plethora of different signaling pathways. Its versatility is achieved by its rich dynamics in concentration changes, which can be caused either by Ca^2+^ entry from the extracellular space or Ca^2+^ release from intracellular storage compartments [2] or by other side pumping Ca^2+^ out of the cell or to intracellular organelles. The main Ca^2+^ storage in mammal cells is, depending on the cell type, the sarcoplasmic–endoplasmic reticulum (SR/ER) [3]. Intracellular concentration of Ca^2+^ is in the range of nM, whereas extracellular Ca^2+^ is in the range of mM [1]. Changes in intracellular Ca^2+^ levels are required for different structures, cell compartments, receptors, channels, Ca^2+^-binding proteins, pumps, transporters, enzymes, and transcription factors [4]. In addition, when intracellular levels rise above physiological concentration, a number of deleterious cellular processes can be triggered [5].

In non-excitable cells, the pathways regulated by these Ca^2+^ signals encompass a wide variety of processes, including from gene expression to fertilization, secretion, protein folding, energy metabolism, and cell cycle regulation [6,7,8]. In excitable cells, the signal depends on Ca^2+^ entry through voltage or ligand-operated channels, which regulates muscle contraction, postsynaptic potentials, memory formation in neurons (long term potentiation), and insulin secretion from beta cells [9].

Due to the huge amount of Ca^2+^-dependent events occurring in cells, alteration of its signaling pathways contributes to the development of multiple human disorders. Therefore, the study of Ca^2+^ signaling is essential for understanding the pathophysiology of many diseases, including diabetes, carcinogenesis, cardio- and cerebrovascular diseases including endothelial dysfunction, as well as neurodegenerative disorders [4,10,11,12,13,14,15,16].

In this review, we describe the importance of Ca^2+^ signaling in mitochondria and how the mechanism of communication between the ER and the mitochondria is pivotal to the mitochondria. Lately, we address different therapeutic strategies targeting mitochondrial Ca^2+^ uptake as an emergent therapeutic approach for neurological disorders.

## 2. MAMs’ Composition and Function

Mitochondria and the ER are structures that experience continuous remodeling to coordinate complex mechanisms of signal transduction and gene expression, generating physical interactions that facilitate a fast and efficient regulation of these processes [17]. Termed endoplasmic reticulum–mitochondria-associated membranes (MAMs), the contact sites between the two compartments are dynamic structures that are highly sensitive to the physiological changes of the cell [18].

The association between the ER and the mitochondria was described in the 1950s, when Copeland and Dalton observed a precise orientation of the ER with respect to the mitochondria [19]. The distance between membranes in this region is 10–30 nm depending on the cell type and cell conditions [20]. Besides, it is estimated that, in physiological conditions, 5–20% of the mitochondrial surface is transiently connected to the ER and these contacts are signaling-dependent [21].

MAMs encompass an extensive variety of different proteins. The first independent proteomic studies identified 911 and 1212 proteins [22,23] localized in the tethers, but only 44% of them were common. During the last decade, different authors have contributed to increment the list by different molecular approaches, such as microscopy or subcellular fractionation [24,25,26]. The development of new techniques has facilitated the proteomic analysis of subcellular domains in-depth. The group of Alice Y Ting has recently identified more than 100 new proteins located in MAMs by means of TurboID technique. This approach was developed to study the interactome of a protein of interest in a specific cell compartment [27]. This emphasizes the complexity of these structures, specialized in each cell type and organism. Indeed, the set of proteins involved in MAMs provides important information about the functions regulated in this domain. As proteins involved in essential cellular processes belong to both the ER and the mitochondrial membranes, the contacts between the organelles enable a coordinated regulation of events, such as lipid biosynthesis [28,29], mitochondrial biogenesis [30,31,32,33], mitochondrial dynamics [34,35], and Ca^2+^ transfer [21,36].

Ca^2+^ exchange between the ER and the mitochondria requires the formation of a protein bridge composed by proteins of both compartments [37]. In particular, the formation of microdomains localized in the ER–mitochondria contact sites promotes a rapid and efficient exchange of Ca^2+^, fundamental for mitochondrial function, dynamics, and the regulation of apoptosis [38]. In 1993, Rosario Rizzuto and colleagues reported the increase in mitochondrial Ca^2+^ upon the cation mobilization through the ER channel IP_3_R (inositol 1,4,5-trisphosphate receptor). Recently, the spatial relation between the ER and the mitochondria was described by the same group [39]. They observed numerous close appositions between these two organelles that contributed to Ca^2+^ entry into the mitochondria in Hela cells [21].

The lumen of the ER is one of the main storages of free Ca^2+^ in the cell (about 100–500 µM) compared to the cytosol (~100 nM). Ca^2+^ is released to the cytosolic space upon the input signals from the ER through the IP_3_R and through the RyR (ryanodine receptor) in the case of the SR [40]. Furthermore, Sig-1R (Sigma non-opioid intracellular receptor 1 or shortly Sigma 1R), located in the ER, is also involved in Ca^2+^ signaling regulation. Sig-1R is enriched in MAMs and stabilizes activated IP_3_R, promoting Ca^2+^ influx into the mitochondria [41,42].

For the mitochondria, Ca^2+^ must cross both mitochondrial membranes. The outer mitochondrial membrane (OMM) is Ca^2+^ permeable due to VDAC (voltage-dependent anion channel), which enables different metabolites (succinate, malate, pyruvate, NADH, ATP, and phosphate) to cross from the cytosol to the mitochondria [36]. In connection with the inner mitochondrial membrane (IMM), Ca^2+^ enters the mitochondrial matrix through the mitochondrial calcium uniporter (MCU) since this layer is ion-impermeable [43].

In addition, another key protein that stabilizes the connections of both compartments is glucose-regulated protein 75 (GRP75), which chaperones IP_3_R and VDAC, maintaining the junction and ensuring an efficient transfer of Ca^2+^ to the mitochondria [36,44]. Altogether, all these attributes highlight MAMs as a coordinated domain that requires an optimal communication between the ER and the mitochondria.

## 3. Ca^2+^ Regulates Mitochondrial Functions

IP_3_R-GRP75-VDAC-MCU is one of the complexes in MAMs that is not only essential for the regulation of Ca^2+^ homeostasis, but also for the control of mitochondrial function in the regulation of bioenergetics, mitochondrial dynamics, and cell death [45,46]. Mitochondria are considered the powerhouse of the cell, providing at least 90% of energy in most cell types. In this context, energy requirements and, thus, mitochondrial function depend on the function of each tissue, as well as developmental and physiological conditions. Mitochondria mainly orchestrate the metabolic profile of tissues with high energy demand, such as heart, liver, kidney skeletal muscle, and brain [47]. In particular, neurons consume 70–80% of the total energy of the brain, being the remaining spent by glial cells [48]. Thus, disruption of bioenergetic pathways compromise mitochondrial function, contributing to pathological features displayed in neurological disorders.

The mechanism of ATP production depends on oxidative phosphorylation, and it is dynamically and promptly regulated by mitochondrial Ca^2+^ levels. Enzymes of the tricarboxylic acid cycle and the electron transport chain require an increase in mitochondrial Ca^2+^ uptake to promote ATP synthesis [49,50]. For instance, isocitrate dehydrogenase and oxoglutarate dehydrogenase are activated upon Ca^2+^ increase in the mitochondrial matrix [51,52]. Furthermore, pyruvate dehydrogenase is a key complex of oxidative metabolism that links glycolysis with the tricarboxylic acid cycle and is influenced by mitochondrial Ca^2+^ concentration. In this way, when energy demand increases, different neurotransmitter and hormone receptors increase mitochondrial Ca^2+^ through IP_3_R [17,53].

Mitochondrial Ca^2+^ uptake is also influenced by mitochondrial dynamics. Several properties of mitochondria, in terms of network, orientation, and shape, regulate the amounts of Ca^2+^ that reach the mitochondrial matrix and, thus, the subsequent functions regulated by these events [54].

1. The first is distribution, because clustered mitochondria are able to buffer Ca^2+^ more efficiently than disperse mitochondria. Mitochondrial fusion/fission events require elevated amounts of cytosolic Ca^2+^ to be transferred to the mitochondria [33,55]. In this way, Ca^2+^ can activate the cytosolic GTPase dynamin-related protein (Drp-1), which is recruited to form a ring around mitochondria to promote mitochondrial fission [31]. On the other hand, mitofusin 2 (MFN2), the GTPase responsible for the OMM fusion, also participates as a key regulator of MAMs, contributing to intracellular Ca^2+^ homeostasis [33].
2. Connectivity, because elongated mitochondria are better Ca^2+^ conductors, distributing the cation along the fused network. It has been described that, while fragmented mitochondria buffer Ca^2+^ from the ER in a heterogeneous manner, tubular mitochondria incorporate Ca^2+^ in an equilibrated and connected way [54,56].
3. Vicinity is a dynamic property that also affects Ca^2+^ buffering, since elevated concentrations of Ca^2+^ near mitochondria are required to promote mitochondrial Ca^2+^ uptake. In this context, Csordás et al. demonstrated the importance of spacing distance in MAMs for an efficient Ca^2+^ transfer, outlining that it is essential that the tethered bridge is properly assembled to ensure Ca^2+^ influx into the mitochondria [44,57].
4. Last but not least, the volume of mitochondria is also crucial for mitochondrial Ca^2+^ buffering, as forced mitochondrial expansion [57] and fragmentation reduce Ca^2+^ uptake capacity [58].

Furthermore, abnormal Ca^2+^ accumulation in the mitochondria normally precedes cell death driven by necrosis and apoptosis [59,60]. Under physiological and pathological conditions, impaired Ca^2+^ handling can lead to mitochondrial Ca^2+^ overload, thus activating the opening mitochondrial permeability transition pore. This causes mitochondrial swelling, which leads to release of cytochrome c and caspase cofactors into the cytosol [60,61].

## 4. Ca^2+^ and Oxidative Stress

Mitochondria is considered the main source of reactive oxygen species (ROS) in cells with high metabolic rates. In circumstances of mitochondrial dysfunction, an uncontrolled production of ROS would lead to an imbalance in the cellular redox state which, in turn, might likely contribute to pathogenesis [62]. Oxidative stress occurs when ROS production exceeds detoxification, causing cell damage. Thus, a balance between ROS production and antioxidant systems is crucial to maintain cell homeostasis and survival [63,64]. The electron transport chain located in mitochondrial cristae generates ROS, such as the superoxide anion (O_2_^−^), which are converted to the diffusible redox signaling molecule hydrogen peroxide (H_2_O_2_) by superoxide dismutase 2 (SOD2). There is mounting evidence about the role of H_2_O_2_ as a messenger located in redox nanodomains in MAMs [65]. H_2_O_2_, mainly generated in the mitochondria, can modulate the activity of IP_3_R and RYR channels, promoting the release of Ca^2+^ from the ER to the mitochondria. This event can, in turn, induce redox signaling through the activation of mitochondrial metabolism, further inducing the accumulation of additional H_2_O_2_. This positive feedback mechanism attenuates when Ca^2+^ returns from the mitochondrial matrix to the ER [66,67]. This process can be beneficial or detrimental, depending on the cellular context and the levels of ROS generated. Indeed, excessive amounts of mitochondrial Ca^2+^ lead to high ROS levels, which may trigger cell death [65]. In addition, it has been reported that mitochondrial Ca^2+^ overload can inhibit H_2_O_2_ clearance, promoting its accumulation in the mitochondria [68].

Hence, this outstands the necessity of an equilibrate Ca^2+^ exchange ER–mitochondria through proper contacts between the two compartments. Nonetheless, a growing knowledge and a better understanding about the translational and clinical role of Ca^2+^ homeostasis and oxidative stress in the physiopathology of neurological diseases are required to find novel therapeutic strategies.

## 5. MAMs’ Communication and Neurological Diseases

As it has been discussed above, the regulation of MAMs is crucial to maintain a proper Ca^2+^ exchange and regulate key mechanisms in cell homeostasis. These processes (lipid metabolism, Ca^2+^ homeostasis, mitochondrial dynamics, and axonal maintenance) are usually involved in the physiopathology of neurodegeneration. Mitochondrial dysfunction results in abnormal Ca^2+^ handling, leading to alterations in axonal transport, bioenergetics, redox status, contractility, and cell viability [69,70,71]. It is clear that ER–mitochondria communication is very important for axonal survival and degeneration. In fact, MAMs are functionally implicated in axons, dendrites, and neuronal soma, modulating and maintaining synaptic activity [72,73,74,75]. For this reason, ER–mitochondria assembly has been proposed as a common mechanism in neurodegenerative disorders [76,77,78,79].

The first proteomic description of MAMs detected proteins involved in mitochondrial dysfunction as well as in neuromuscular and degenerative diseases, including Huntington’s disease (HD), Parkinson’s disease (PD), and Alzheimer’s disease (AD) [23]. Nowadays, it is well described that the proteins involved in these diseases are resident in MAMs [80,81,82,83]. Specifically, these diseases exhibit increased ER–mitochondria contacts, leading to toxic mitochondrial Ca^2+^ uptake, reduced cell viability, and dysfunction in the subsequent mechanisms regulated in this domain [77,78,79].

Conversely, several neuropathies exhibit reduced ER–mitochondria connections and Ca^2+^ accumulation in the cytosol, impairing the communication between the two compartments and displaying mitochondrial alterations. Several forms of amyotrophic lateral sclerosis (ALS) are characterized by mitochondrial disruption and abnormal mitochondrial Ca^2+^ handling. This includes ALS forms with mutations in superoxide dismutase 1 (SOD1), in which mouse models exhibit mitochondrial defects and deficits in mitochondrial Ca^2+^ uptake [84,85]. Homozygous mutations in the ER protein Sig-1R are the cause of the juvenile form of ALS16 [86], showing ER–mitochondria dissociation in motor neurons, as well as a reduction in mitochondrial Ca^2+^ influx via IP_3_R, and lower ATP production. These defects lead to neuron vulnerability, directly associated with the physiopathology of the disease [87].

Accordingly, dominant cerebellar ataxias are multifactorial and progressive diseases with common mechanisms of mitochondrial dysregulation, Ca^2+^ handling defects, and oxidative stress that contribute to neurodegeneration [88]. Specifically, spinocerebellar ataxias type 2 and 3 (SCA2/3) have been found to exhibit mutations in the IP_3_R channel, which leads to the abnormal Ca^2+^ release to the cytoplasm, potentially inducing Ca^2+^ buffering defects in mitochondria [89]. Interestingly, different models of the recessive neuromuscular disorder Friedreich’s ataxia (FRDA) exhibit cytosolic Ca^2+^ accumulation, as well as impaired mitochondrial Ca^2+^ uptake and decreased inter-organelle interactions in MAMs. The protein involved in the disease, frataxin, has recently been found as a member of the protein network of MAMs, interacting with GRP75 and IP_3_R [90,91,92]. Charcot–Marie–Tooth (CMT) is an inherited neuropathy caused by mutations in an important number of proteins related to mitochondrial function. Specifically, the causative genes for CMT type 2 are ganglioside-induced associated protein 1 (*GDAP1*) and *MFN2* [93], which encode proteins located in the OMM that contribute to MAM’s function. Deficiency in GDAP1 leads to neuronal Ca^2+^ and mitochondrial defects, coupled with altered interplay between ER–mitochondria and Ca^2+^ accumulation in the cytosol [71,94,95]. Hereditary spastic paraplegia is associated with alterations in genes related to the ER that also affect the axonal transport of mitochondria, including IP_3_R, suggesting impaired ER–mitochondria communication [96,97,98].

## 6. Therapeutic Approaches Targeting MAMs

**Figure 1:**
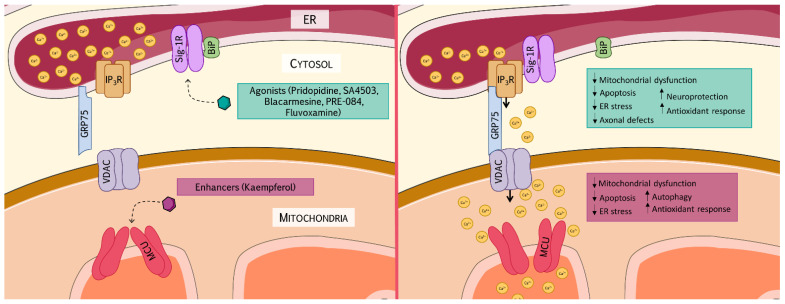
Schematic representation showing the effects of mitochondrial Ca^2+^ uptake promoters. The ER is the main Ca^2+^ storage in the cell and Ca^2+^ exchange between the ER and the mitochondria requires the formation of tethers composed by proteins of both compartments. Sig-1R resides in the ER membrane, in a dormant, Ca^2+^-dependent state. Upon activation by agonists, Sig-1R dissociates from BiP/GRP78 and reallocates within the ER membrane, interacting with IP_3_R and chaperoning the protein complex that transfers Ca^2+^ to the mitochondria. This complex formed by IP_3_R-GRP75-VDAC ensures a rapid Ca^2+^ flux to the mitochondrial intermembrane space, which triggers MCU opening and Ca^2+^ to cross the mitochondrial inner membrane. Several models of neurological disorders such as ALS, CMT and FRDA have exhibited alterations in mitochondrial Ca^2+^ buffering by defective appositions between the two organelles. Both Sig-1R agonists and MCU enhancers promote Ca^2+^ exchange between the ER and the mitochondria, exerting beneficial effects in different models of neurological diseases. On the one hand, Sig-1R agonists (pridopidine, SA4503, Blacarmesine, PRE-084, and fluvoxamine) have been demonstrated to exert neuroprotective effects, improving mitochondrial dysfunction, preventing cells from apoptosis, activating the antioxidant response, ameliorating ER stress, and improving axonal defects. On the other hand, the MCU enhancer, Kaempferol, has helped to improve mitochondrial dysfunction, activate the oxidative stress response, modulate autophagy, regulate ER stress, and prevent cells from apoptosis. This figure has been created using Creative Commons resources from Servier Medical Art [99]. ALS: amyotrophic lateral sclerosis; Bip/GRP78: binding immunoglobulin protein/glucose-regulated protein 78; CMT: Charcot–Marie–Tooth; ER: endoplasmic reticulum; FRDA: Friedreich’s ataxia; GRP75: glucose-regulated protein 75; IP_3_R: inositol 1,4,5-trisphosphate receptor; MCU: mitochondrial calcium uniporter; Sig-1R: sigma non-opioid intracellular receptor 1; VDAC: voltage-dependent anion channel.

Mitochondrial Ca^2+^ modulation is fundamental to maintain the physiological mechanisms that regulate metabolism, mitochondrial dynamics, and cell death. Therefore, MAMs emerge as potential therapeutic targets of neurological disorders. In this review we will focus on different therapeutic approaches (see Figure 1 for further information [99]) aimed to stabilize the ER–mitochondria assembly and, thus, promote Ca^2+^ influx into the mitochondria.

### 6.1. Sigma-1 Receptor as a Therapeutic Target

Sig-1R is a Ca^2+^-sensitive chaperone located in the ER membrane, specifically in MAMs, and regulates Ca^2+^ homeostasis, lipid dynamics, MAMs’ stability, and the ER stress response [41,100]. Sig-1Rs of different species share a very high sequence identity (>93%), but share no sequence homology with any other mammalian protein. Interestingly, the Sig-1R displays a 30% identity, a 67% homology, and a similar ligand profile to the yeast sterol isomerase encoded by the *ERG2* gene [101,102]. This conservation points towards Sig-1R as a fundamental protein for cell functioning. It is highly expressed in the central nervous system, playing a key role in physiological functions, such as cell differentiation, axon formation, microglial activation, and astrocyte regulation [103].

Sig-1R resides specifically on ceramide and cholesterol-rich lipid microdomains at MAMs, acting as an inter-organelle Ca^2+^ signaling modulator and exerting a pivotal role in neuroprotection and neuroplasticity [104]. In physiological conditions, Sig-1R forms a complex with the chaperone binding immunoglobulin protein/glucose response protein 78 (BiP/GRP78), in a dormant, Ca^2+^-dependent state. Once activated by agonists or ER Ca^2+^-depletion, Sig-1R dissociates from BiP and reallocates within the ER membrane, interacting with different proteins, such as IP_3_R. Then, IP_3_R is prevented from degradation and promotes mitochondrial Ca^2+^ uptake (see Figure 1 for further information [99]) [42]. Thus, Sig-1R is a key element for maintaining the structure and function of MAMs. Besides, during ER stress response, Sig-1R expression increases, which prevents cells from apoptosis triggered under such conditions [105]. Sig-1R has been recently identified as indispensable for mitochondrial bioenergetics during early ER stress, which gives rise to an increased ER–mitochondrial Ca^2+^ exchange [106]. Hence, targeting Sig-1R may regulate ER stress, a common mechanism displayed in neurodegeneration [107]. Interestingly, Sig-1R ligands have been found to potentially offer protection against the most severe symptoms of SARS-CoV-2, providing mitochondrial protection, activating mitophagy, preventing ER-stress, managing Ca^2+^ transport, and inducing autophagy to prevent cell death in response to infection [108].

Recently, the basic structural pharmacophore of Sig-1R has been identified, which is critical for drug development. There is evidence about different shifting monomerization–oligomerization states of Sig-1R modulated by its ligands. In this sense, agonists and antagonists regulate the association between Sig-1R and BiP, controlling the interactome of Sig-1R [105,109,110]. The regulation of these mechanisms has a pivotal role in the context of MAMs and neurodegenerative diseases [111]. Transcriptional upregulation of Sig-1R induces its neuroprotective properties. For instance, it has been reported that Sig-1R upregulates the expression of the antiapoptotic mitochondrial protein Bcl-2 (B-cell lymphoma 2), preventing neuronal cell death [112]. On the other hand, Sig-1R has been involved in the protection of cellular oxidative stress and the activation of antioxidant response elements [113]. In this line, overexpression of Sig-1R enhanced resistance to oxidative stress in *Drosophila melanogaster* [114]. Conversely, knockdown of Sig-1R leads to increased ROS and decreased expression and activity of nuclear factor erythroid 2-related factor 2 (NRF2), the protein implicated in the activation of the intracellular antioxidant response [115]. These data point Sig-1R as a potential therapeutic target against oxidative stress-related diseases.

While Sig-1R deficiency exacerbates progression of neurological disorders and symptoms commonly associated to neurodegenerative disorders [116,117,118], many Sig-1R agonists exert anti-amnestic, synaptogenesis, and neuroprotective effects under neuronal stress conditions [119,120]. Indeed, the absence of Sig-1R leads to motor neuron degeneration, associated with reduced ER–mitochondria contacts, disturbed mitochondrial dynamics, and intracellular Ca^2+^ dyshomeostasis [121]. Remarkably, pharmacological modulation of Sig-1R has been demonstrated to mitigate disease and symptoms in different models of ALS, AD, PD, and HD (reviewed in [105]). Given the chaperone nature of Sig-1R, its activity in targeting conformationally misfolded proteins occurs only under such conditions. Indeed, it has been described that Sig-1R activation only exhibits therapeutic effects under pathological conditions and has no effect in control animals [122,123]. This provides evidence about the specificity of Sig-1R ligands in pharmacotherapy.

#### 6.1.1. Pridopidine

Pridopidine (ACR16) is a selective Sig-1R agonist and a dopamine stabilizer [124,125]. Great focus has been given to pridopidine in recent years, especially in HD, where it has demonstrated to improve motor function in both animal models and patients, protecting neurons from mutant huntingtin toxicity [126]. For instance, Ryskamp and colleagues evaluated the relevance of Sig-1R as a therapeutic target of pridopidine in HD. Action of pridopidine is mediated by Sig-1R, leading to restoration of IP_3_R-dependent Ca^2+^ release and upregulation of key Ca^2+^-regulating genes [126]. Furthermore, early treatment of pridopidine prior to the appearance of disease phenotypes improved motor coordination and reduced anxiety and depressive-like phenotypes in the YAC128 HD mice model, whereas late treatment only rescued depressive-like symptoms [126]. Interestingly, the research carried out by Naia et al. using different models of HD has highlighted the effects of pridopidine in mitochondrial function, contributing to neuroprotection mediated by Sig-1R. Authors reported that pridopidine prevented the disruption of ER–mitochondria contacts, also improving the colocalization of IP_3_R and Sig-1R with mitochondria in YAC128 neurons. Accordingly, this compound increased mitochondrial activity, elongation, and motility. In both HD human neural stem cells and YAC128 neurons, pridopidine increased mitochondrial respiration, rescued antioxidant response, and decreased mitochondrial ROS levels caused by Sig-1R knockdown. Additionally, apart from the improvement in motor coordination, YAC128 mice treated at early/pre-symptomatic age with pridopidine showed a reduction in mitochondrial ROS levels. Overall, these results highlighted the effects of pridopidine in mitochondrial function, contributing to neuroprotection mediated by Sig-1R [126].

Regarding clinical evaluation, pridopidine has demonstrated some improvements in motor symptoms. A phase-III randomized, double-blind, multicenter trial study (NCT00665223, MermaiHD) failed to provide evidence about the effects of pridopidine in the primary outcome (changes in modified Motor score). Despite this, some parameters related to motor scales improved significantly in the 90 mg/day group [127]. According to these results, a randomized, double-blind, placebo-controlled, multicenter trial (HART study, NCT00724048) could not reach its primary endpoint after 12 weeks of treatment. Nevertheless, patients treated with the highest doses improved in secondary motor evaluation, suggesting modest beneficial effects of pridopidine in HD [127]. Altogether, these results suggest the reproducible positive effects of pridopidine on motor symptoms in HD. Currently, a phase-III, randomized, double-blind, placebo-controlled study is recruiting patients to evaluate the efficacy and safety of pridopidine in patients with early-stage HD (NCT04556656) [128].

The efficacy of pridopidine has been tested in in vitro and in vivo models of ALS, due to the fact that different forms of ALS are caused by mutations in the Sig-1R gene [87,129]. Authors demonstrated beneficial effects of pridopidine-targeting Sig-1R on axonal transport perturbations, neuromuscular junction disruption, and motor neuron death. In addition, pridopidine slowed the progression of the disease in an ALS mouse model [130]. There is currently an ongoing phase-II clinical trial on the efficacy of pridopidine in ALS patients (NCT04615923) [131].

Recently, pridopidine has demonstrated neuroprotective and neurorestorative effects in nigrostriatal dopamine neurons via Sig-1R in an animal model of PD [132]. A phase-II, double-blind, parallel-group study started in 2019 with the aim of assessing two doses of pridopidine in levodopa-induced dyskinesia patients with PD (gLIDE study, NCT03922711) [133]. Despite the pending results, it seems that the study was cancelled early due to COVID-19 pandemic.

#### 6.1.2. SA4503

SA4503 or cutamesine is an orally available, potent, and selective Sig-1R agonist that exhibits antiarrhythmic and antidepressant effects [134]. It has been reported to alleviate mitochondrial dysfunction, recovering ATP production in a dose-dependent manner and mobilizing intracellular Ca^2+^ into the mitochondria. These effects attenuated neuronal apoptosis [135] and ameliorated cardiac hypertrophy [136]. In addition, SA4503 promoted the survival of cortical neurons from oxidative-stress-induced cell death [137]. In ALS models, this compound has shown to suppress motor neuron degeneration and symptom progression [137]. In this line, SA4503 also enhanced the cytosolic Ca^2+^ clearance in motoneurons and IP_3_R-mediated ER Ca^2+^ release in ALS mice [138]. In alpha-thalassemia X-linked intellectual disability, SA4503 reversed axonal development and dendritic spine abnormalities in cultured cortical neurons, as well as cognitive deficits exhibited in the mice model [139].

In the clinical field, SA4503 has been evaluated in two trials. In 2008, the safety and efficacy of SA4503 was assessed in subjects with major depressive disorder (NCT00551109) [140]. Results and outcomes are pending. Furthermore, a phase-II, double-blind, placebo-controlled, ascending dose study evaluated the safety and motor function restoration in subjects with acute ischemic stroke (NCT00639249). Even though it was safe and well tolerated, no significant improvements were found [139].

#### 6.1.3. Blarcamesine

Blarcamesine (ANAVEX2-73) is a safe Sig-1R agonist and muscarinic receptor modulator with preliminary efficacy evidence in patients with AD and Rett syndrome [141]. Preclinically, blarcamesine exerted anticonvulsant, anti-amnesic, neuroprotective, and antidepressant effects in various animal models of Rett syndrome [141], fragile X syndrome [141], AD [142,143], and amnesia [144]. These data suggest its potential in neurodegenerative and neurodevelopmental diseases.

After demonstrating good safety, bioavailability, and tolerability in AD patients (NCT02244541) [145], blarcamesine has been tested in a phase-II, placebo-controlled study with Rett Syndrome patients (NCT03758924) [146]. Even though the trial is completed, results are still pending. Currently, different phase-II/III trials focused on PD (NCT04575259) [147], AD (NCT04314934) [148], and Rett Syndrome (NCT03941444, NCT04304482) [149,150], which are recruiting patients to evaluate the tolerability and efficacy of blarcamesine.

#### 6.1.4. PRE-084

PRE-084 is a selective Sig-1R agonist that has demonstrated promising effects against oxidative stress and modulating intracellular Ca^2+^ levels in preclinical studies using different disease models. For instance, PRE-084 exerted protective action against oxidation and improved viability in human retinal cells [151]. In a HD cell model, pre-treatment with PRE-084 resulted in the prevention of caspase 3-cleavage, stimulation of cellular antioxidants, and a reduction in ROS in mutant huntingtin-expressing neuronal cells [152].

Furthermore, in a model of AD, treatment with PRE-084 restored mitochondrial respiratory dysfunction in mouse hippocampus and prevented increases in lipid peroxidation levels and apoptosis markers [143]. In this line, Watanabe S. et al. evaluated a Ca^2+^ influx into the mitochondria and ATP levels after incubating motor neurons of ALS with PRE-084. In all the experiments, this compound restored the function of IP_3_R impaired in the model, suggesting that Sig-1R activation by PRE-084 prevented the disruption of Sig-1R-IP_3_R interaction. In addition, intraperitoneal administration of PRE-084 in pre-symptomatic ALS mice successfully restored co-localization of Sig-1R and IP_3_R analyzed in neurons of the lumbar spinal cord. These data indicate that Sig-1R activation is crucial to prevent MAMs disruption and regulate the Ca^2+^ exchange between the ER and the mitochondria via IP_3_R [87]. PRE-084 improved locomotor function and motor neuron survival in pre-symptomatic and early symptomatic mutant ALS mice. Authors reported a promising strategy of pharmacological manipulation of Sig-1R, pointing to an increased availability of growth factors, as well as modulation of astrocytosis and macrophage–microglia as part of the mechanisms involved in Sig-1R-mediated neuroprotection [87]. Nevertheless, one of the studies aforementioned about the treatment of ALS motor neurons with SA4503 also tested PRE-084. Contrary to SA4503, PRE-084 did not reduce the cytosolic Ca^2+^ levels [138]. These findings indicate that different Sig-1R ligands may have different effects on MAMs-regulated Ca^2+^ homeostasis.

#### 6.1.5. Fluvoxamine

Fluvoxamine is a selective serotonin reuptake inhibitor with high affinity for Sig-1R that has been widely used in clinical practice as an antidepressant. Fluvoxamine has shown to increase Sig-1R expression, inducing neuroprotection and protecting cells from ER-stress [153]. This compound has also been found to rescue impaired mitochondrial Ca^2+^ uptake and ATP production in hypertrophic cardiomyocytes [154] and protect against cardiac dysfunction [155]. Furthermore, activation of Sig-1R by fluvoxamine has been demonstrated to activate several antioxidant pathways [155,156]. For instance, in brain and liver of oxidative stress-induced mice, fluvoxamine alleviated lipid peroxidation and oxidative stress by reducing malondialdehyde and nitric oxide levels and increasing reduced glutathione (GSH) [157]. Accordingly, this drug exerted anti-inflammatory and antioxidant properties by enhancing GSH levels and reducing the nitric oxide-dependent oxidative marker 3-nitrotyrosine [158]. Special attention has been paid lately to fluvoxamine regarding COVID-19. Clinically, this compound is well tolerated and widely available. Its mechanisms of action are involved with the hallmarks of severe COVID-19 (reviewed in [159]). At the moment, fluvoxamine has not been used in the clinical field beyond antidepressant or COVID-19 purposes.

### 6.2. Mitochondrial Calcium Uniporter as a Therapeutic Target

The mitochondrial calcium uniporter (MCU) is one of the most important and highly selective Ca^2+^ transporting complexes [160]. MCU is a crucial element of MAMs, acting as a gatekeeper of Ca^2+^ and controlling the mitochondrial Ca^2+^ influx. In fact, it has been determined that MCU and IP_3_R must have optimal distance to ensure mitochondrial Ca^2+^ signaling in physiological conditions [161]. Located in the IMM, this pore-protein is composed of several subunits. While the pore-forming and Ca^2+^-conducting subunit of the MCU complex is named Mcu, the regulatory subunit encompasses MICU1; MICU2; mitochondrial calcium uptake protein 1, 2, 3 (MICU3); essential MCU regulator (EMRE); mitochondrial calcium uniporter regulator 1 (MCUR1); and mitochondrial calcium uniporter regulatory subunit (MCUb) [162,163].

Specifically, MICU1 is an important gatekeeper located towards the mitochondrial matrix, next to the IMM. MICU1 controls the mitochondrial Ca^2+^ influx by interacting with Mcu, so when binding to Ca^2+^, its conformation changes from hexamers to oligomers and activates MCU [164,165]. Knock out of MICU1 in mice causes significant mortality, marked ataxia, and muscle weakness. Furthermore, patients with mutations in MICU1 exhibit brain and muscle disorders, proximal myopathy, learning difficulties, and a progressive extrapyramidal movement disorder [166]. Besides, MICU2 is also a gatekeeper and exhibits an inhibitor role of Ca^2+^ uptake, preventing Ca^2+^ to cross the IMM unless it is above the threshold. In fact, elevated Ca^2+^ levels in the intermembrane space are required to reach the mitochondrial matrix through MCU due to its low Ca^2+^ affinity [167]. MICU3 displays the same role as MICU2 but, in comparison, has a different tissue-dependent expression pattern [168]. EMRE is a single-pass transmembrane protein that functions as a positive regulator of MCU, and its interaction with MCU is essential for mitochondrial Ca^2+^ uptake, acting as a gatekeeper and preventing mitochondrial Ca^2+^ overload [169,170]. MCUR1 exerts a scaffold role, binding Mcu and EMRE [171,172]. MCUb is the negative regulatory subunit of MCU since its overexpression leads to a decreased Ca^2+^ intake by the MCU [173].

In the last decade, special interest has been taken in the modulation of MCU activity as a novel therapeutic target. Most current therapeutic approaches are focused on the inhibition of ER–mitochondria Ca^2+^ exchange, mainly in cancer [174,175] and neurodegenerative disorders [176] (such as AD [177] and PD [178]).

However, diseases such as type 2 diabetes point towards MCU activation to alleviate mitochondrial dysfunction associated with dysregulation in intracellular Ca^2+^ homeostasis, MAMs disruption, and defects in several functions regulated in this domain [179,180]. Targeting the MCU has been demonstrated to be beneficial in models with impaired ER–mitochondria inter-organelle communication. For instance, MCU activation by spermine increased cytosolic Ca^2+^ clearance by promoting mitochondrial Ca^2+^ buffering capability in cardiomyocytes with cardiac hypertrophy. In this cellular model, SR–mitochondria connections were decreased by the inhibition of Mfn2, leading to inhibition of ATP synthesis and contributing to pathogenesis [181].

Besides, genetic modulation of MCU expression has proved to modulate mitochondrial Ca^2+^ uptake [182]. In this line, either MCU overexpression or MCU activation by spermine reversed Pb^2+^-induced oxidative stress and inhibition of mitochondrial Ca^2+^ uptake in SH-SY5Y human neuroblastoma cells [183]. The promotion of mitochondrial Ca^2+^ import through MCU overexpression in glia recovered degenerative phenotypes and ATP production in a FRDA *Drosophila melanogaster* model [90].

#### Kaempferol

The most relevant MCU enhancer described until now is Kaempferol, which has demonstrated to enhance MCU, promoting Ca^2+^ import into the mitochondria and exhibiting neuro and cardioprotective properties [184,185,186,187,188]. In 2004, Montero and collaborators identified different flavonoids with the effect of stimulating mitochondrial Ca^2+^ entry through MCU activation. The most active compound was Kaempferol, which increased the rate of mitochondrial Ca^2+^ uptake by 85-fold [189]. In addition, Kaempferol modulates autophagy to protect cells from malfunction and regulates ER stress [190,191]. Special attention has been paid to Kaempferol recently due to its anti-inflammatory and antioxidant properties. For instance, Kaempferol was able to induce NRF2 expression in brain tissues [184]. A recent study suggests that Kaempferol augmented the phosphorylation of PIK3 and Akt, thus allowing Keap-1 to release NRF2 and promote antioxidant response in the nucleus [192].

Accordingly, in cerebellar granule cells, Kaempferol prevented cells from apoptosis, exerting potent effects by blocking ROS production [193]. In ischemia–reperfusion injury models, Kaempferol reduced mitochondrial dysfunction and reduced oxidative stress by decreasing ROS and malondialdehyde levels, whereas GSH and GSH peroxidase levels increased significantly [194,195,196]. In a *D. melanogaster* model of PD, kaempferol delayed degenerative phenotype onset in a dose-dependent manner, accompanied by a reduction in oxidative stress markers [197]. Similar effects on phenotype and oxidative stress were reported in a *D. melanogaster* model of AD [198]. Indeed, a study conducted with 921 participants concluded that kaempferol and other flavonoids are associated with lower risk of developing AD [199]. Kaempferol was proposed as a bone-fide candidate for the design of therapeutic approaches against familial ALS from a computational perspective through molecular docking, quantum chemical studies, and molecular dynamics [200].

### 6.3. Other Approaches

In addition to the aforementioned approaches, other compounds may be suitable to increase mitochondrial Ca^2+^ uptake without a specific ER–mitochondria target.

#### 6.3.1. Taurine

Taurine is a sulfur-containing amino acid present in abundance in many excitable tissues, including the brain, skeletal, and cardiac muscles. Physiological actions of taurine include membrane stabilization, neurotransmission, and modulation of cellular Ca^2+^ levels [201]. El Idrissi and Trenkner evaluated the neuroprotective role of taurine in the regulation of mitochondrial Ca^2+^ buffering in cerebellar granule cells. They demonstrated an active role in the regulatory mechanisms of Ca^2+^ homeostasis, suggesting an enhancement in mitochondrial function and regulation of intracellular Ca^2+^ [202,203]. Besides the suggestion of a selective mechanism of taurine in mitochondrial Ca^2+^ uptake enhancement, though the specific receptor of taurine is not known.

#### 6.3.2. Nerve Growth Factor

Nerve growth factor (NGF) is a secreted neurotrophin involved in survival, maintenance, and regeneration of specific types of neurons in the central and peripheral nervous system [204]. It has been highlighted as a potential therapeutic option for neurodegeneration due its role in apoptosis prevention [205], mitochondrial dysfunction protection [206], mitochondrial remodeling, and intracellular Ca^2+^ mobilization [207,208]. In the context of Ca^2+^ signaling, NGF has been demonstrated to increase cytosolic free Ca^2+^ concentration in C6-2B glioma cells and PC12 cells [209], as well as mitochondrial Ca^2+^ [207]. NGF was assessed in an open-label, dose-escalation study of encapsulated cell biodelivery of NGF in AD patients. Authors reported that preliminary data of the NGF treatment seemed to slow the rate of atrophy depending on the subtype of AD [210]. For this reason, NGF treatment should be further investigated for neuronal support.

#### 6.3.3. MiCUps

Mitochondrial Ca^2+^ uptake enhancers (MiCUps) are a group of compounds recently identified to increase mitochondrial Ca^2+^ import, especially in cardiomyocytes. Using molecular ligand–protein docking and mutational analysis, a recent study has determined Efsevin as a MiCUp that shifts the opening of the VDAC2 channel, promoting Ca^2+^ entry to the mitochondria [211]. It has been mostly used in cardiac models, due to its ability to regulate cardiac rhythmicity [187,212]. Ezetimibe and disulfiram have been recently identified as MiCUps, proving to efficiently suppress arrhythmogenesis in different experimental models. The identification of such compounds underscores mitochondrial Ca^2+^ uptake as a pharmacological target [213].

#### 6.3.4. Antioxidants

Targeting the mitochondrial redox state could be a suitable therapeutic strategy to recover ER–mitochondria communication. In this context, compounds may modulate mitochondrial Ca^2+^ signaling to stabilize its redox state or directly target mitochondrial ROS [214]. The first strategy has been discussed before; many of the aforementioned compounds exert antioxidant properties in addition to its main mechanism of action, which can readjust the ER–mitochondria Ca^+2^ flux and, thus, ensure a correct balance in the ROS–antioxidant system. In the context of the second strategy, treatment with antioxidants has been demonstrated to recover ER–mitochondria communication and Ca^2+^ exchange between the two compartments. Vitamin E restored mitochondrial Ca^2+^ uptake in a cardiomyocyte model of FRDA [91]. Accordingly, trolox (mimic of vitamin E) and N-acetylcysteine were able to recover both function and structure of MAMs in a neuronal model of FRDA [90], suggesting an oxidative environment in MAMs is implied in the pathophysiology of the disease. In essence, the use of antioxidants may be a good strategy to both reduce redox environment in MAMs and potentiate Ca^2+^ influx in the mitochondria.

On the other hand, when mitochondrial function is impaired, it is realistic to assume the involvement of different effectors and mechanisms. For this reason, some authors point towards the use of a combination of antioxidants also known as ‘mitochondrial cocktails’, due to its irrelevant toxicity, providing relevant benefits by increasing the spectrum of action [215,216,217,218].

SS-31 or elamipretide is an aromatic–cationic tetrapeptide that readily penetrates cell membranes and transiently localizes to the inner mitochondrial membrane. This mitochondrial-targeted agent was found to interact with cardiolipin, an anionic phospholipid located in the IMM and required for cristae formation. In addition, SS-31 accelerates ATP recovery and increases the enzymatic activities of Fe-S enzymes, including aconitase and complex II and III of the respiratory chain [219,220]. Another study suggested that SS-31 could have therapeutic potential effects in preventing damage from oxidative stress in neurocognitive disorders [221]. SS-31 has demonstrated to be safe and well tolerated. It has been tested in a crossover clinical trial evaluating its efficacy in primary mitochondrial myopathy for 4 weeks. Patients experienced a clinically meaningful change in the primary endpoint, which was not significant [222]. Nonetheless, these and other relevant results provided efficacy to support the initiation of a 6-month-long, phase-III study. Another clinical trial (NCT04689360) is currently recruiting patients with genetically confirmed rare diseases with known mitochondrial dysfunction [223].

Mitoquinone or MitoQ is an orally active mitochondria-targeted antioxidant that mimics the role of the endogenous mitochondrial antioxidant coenzyme Q10 [224]. In addition, MitoQ has demonstrated to substantially increase the antioxidant capacity of coenzyme Q10 by modulating oxidative stress via activating the NRF2 pathway [225,226] and increasing GSH levels [227]. Treatment in vitro and in vivo with MitoQ has provided evidence about its beneficial effects in neuroprotection, as well as in restoring mitochondrial dynamics, bioenergetics, and the redox state [228,229]. MitoQ is currently being evaluated in several clinical trials, including its assessment in AD (NCT03514875) [230], vascular function (NCT02966665) [231], and multiple sclerosis (NCT04267926) [232], among others.

These and other antioxidants targeting mitochondrial dysfunction in neurodegeneration have been extensively reviewed by several authors, including us [218,233,234,235,236,237,238].

## 7. Conclusions and Future Perspectives

Since mitochondria are intracellular dynamic compartments involved in multiple mechanisms, the in-depth study of these mitochondrial-dependent pathways is crucial to understand the pathophysiology of neurological and neuromuscular disorders. Particularly, we highlight the importance of crosstalk communication between the ER and the mitochondria in intracellular Ca^2+^ homeostasis and, therefore, in cell physiology. Since MAMs are the structures carrying out such communication, their disruption involves dramatic consequences that not only affect mitochondrial mechanisms, but also a plethora of intracellular signaling pathways. An example of such disarrangement is exerted in neurological disorders, as the proteins involved in these diseases are part of the protein network of MAMs [86,90,239].

On the other hand, proteins belonging to MAMs are a growing list, which contributes to the idea of the complexity and dynamism of these structures and, thus, the mechanisms involved in its regulation. For this reason, it is also important to determine the properties of MAMs in different cell types under distinct cellular conditions. The elucidation of these pathways will provide valuable information about the physiopathology of diseases that present impaired ER–mitochondria communication, opening new fields of research to identify adequate treatments for patients.

We believe that restoration of MAMs communication may be a suitable strategy to reverse this impairment. In addition, some patients suffering from neuromuscular diseases usually undergo heart conditions [240,241,242]. Interestingly, the activation of targets, such as Sig-1R, has demonstrated to have both neuro and cardioprotective effects. As compounds such as pridopidine and blacarmesine are currently being evaluated in clinical trials, the results obtained may be applicable to other diseases with common impaired mechanisms. Furthermore, oxidative stress is a common hallmark in neurological disorders [234,243,244], so the activation of antioxidant mechanisms has always been a common therapeutic strategy. The fact that many compounds targeting mitochondrial Ca^2+^ uptake can, therefore, exert antioxidant properties, making them more versatile in restoring the molecular defects involved in these diseases.

In terms of future therapeutic approaches, special attention is being paid to miRNAs. These small molecules regulate genetic expression by binding to its target mRNA and can be detected in biological fluids. The identification of miRNA signatures could provide valuable information about diagnosis or prognosis, even in the early stages of the disease. Besides, this should address the nature of targets and the extent of the biological regulation of the occurring pathways. For instance, miR-20b has been found to mediate MFN2 signaling, which can impair ER–mitochondria Ca^2+^ crosstalk and contribute to cardiac hypertrophy [181]. This opens a new field of therapy development in MAMs communication.

Neurological diseases, such as ALS, CMT, and FRDA, have no cure, so it is essential to find therapeutic approaches that can improve patient’s wellbeing by either slowing the onset of symptoms and/or delaying the progression of the disease, in addition to physical therapy, occupational therapy, and surgery. The repurposing of compounds outlined in this review could be a good strategy to test in vitro and in vivo models of disease. Furthermore, the use of new in silico approaches, such as pharmacophore modeling and molecular docking, are useful to ensure optimal molecular interactions with a specific biological target. This may contribute to identifying new targets, drug discovery, and optimization for specific treatments [245,246,247].

## Data Availability

Not applicable.

## Abbreviations

AD—Alzheimer’s disease	ALS—Amyotrophic lateral sclerosis
Bcl-2—B-cell lymphoma 2	BiP/GRP78—Binding immunoglobulin protein/Glucose response protein 78
Ca^2+^—Calcium	CMT—Charcot–Marie–Tooth
Drp-1—Dynamin -related protein	EMRE—Essential MCU regulator
FRDA—Friedreich’s ataxia	GDAP1—Ganglioside-induced associated protein 1
GRP75—Glucose-regulated protein 75	GSH—Glutathione
H_2_O_2_—Hydrogen peroxide	HD—Huntington’s disease
IMM—Inner mitochondrial membrane	IP_3_R—Inositol 1,4,5-trisphosphate receptor
MAMs—Endoplasmic reticulum–mitochondria-associated membranes	MCU/Mcu—Mitochondrial calcium uniporter
MCUb—Mitochondrial calcium uniporter regulatory subunit	MCUR1—Mitochondrial calcium uniporter regulator 1
MFN2—Mitofusin 2	MICU1/2/3—Mitochondrial calcium uptake protein 1, 2, 3
MiCUps—Mitochondrial Ca^2+^ uptake enhancers	NGF—Nerve Growth Factor
NRF2—Nuclear factor erythroid 2-related factor 2	OMM—Outer mitochondrial membrane
PD—Parkinson’s disease	ROS—Reactive oxygen species
RyR—Ryanodine receptor	SCA2/3—Spinocerebellar ataxias type 2 and 3
Sig-1R—Sigma non-opioid intracellular receptor 1	SOD1—Superoxide dismutase 1
SOD2—Superoxide dismutase 2	SR/ER—Sarcoplasmic/endoplasmic reticulum
VDAC—Voltage-dependent anion channel	
